# Supplementary material for: Mechanisms governing the pioneering and redistribution capabilities of the non-classical pioneer PU.1
Source: Nat Commun. 2020 Jan 21;11:402. doi: 10.1038/s41467-019-13960-2 (PMC6972792; doi:10.1038/s41467-019-13960-2)
Supplement: Supplementary file 7 — Source data [file 41467_2019_13960_MOESM7_ESM.zip › Source_Data/Figure6/Figure6B_MotifScanOutput/FLI1_mutPU.1/homerResults/motif26.similar.html]

motif26

## Information for motif26

A
C
G
T
A
T
C
G
T
C
G
A
G
A
T
C
C
A
T
G
A
G
C
T
T
C
G
A
C
G
T
A
A
G
T
C
  
Reverse Opposite:  

T
C
A
G
G
C
A
T
A
G
C
T
C
T
G
A
G
T
A
C
C
T
A
G
A
G
C
T
T
A
G
C
T
G
C
A
  

|  |  |
| --- | --- |
| p-value: | 1e-44 |
| log p-value: | -1.020e+02 |
| Information Content per bp: | 1.630 |
| Number of Target Sequences with motif | 732.0 |
| Percentage of Target Sequences with motif | 7.13% |
| Number of Background Sequences with motif | 1421.9 |
| Percentage of Background Sequences with motif | 3.73% |
| Average Position of motif in Targets | 95.5 +/- 56.8bp |
| Average Position of motif in Background | 97.4 +/- 62.4bp |
| Strand Bias (log2 ratio + to - strand density) | 0.1 |
| Multiplicity (# of sites on avg that occur together) | 1.04 |
| Motif File: | file (matrix) reverse opposite |

### Similar de novo motifs found

|  |  |  |  |  |  |  |  |
| --- | --- | --- | --- | --- | --- | --- | --- |
| Rank | Match Score | Redundant Motif | P-value | log P-value | % of Targets | % of Background | Motif file |
| 1 | 0.780 | C A T G T G C A A C G T A C T G G C T A A G T C C T A G G C A T G T A C C G T A | 1e-39 | -91.623870 | 7.89% | 4.45% | motif file (matrix) |
| 2 | 0.755 | T C A G A G C T A T G C C G T A G A T C C T A G A C G T A T C G C T G A A G T C A T C G | 1e-29 | -68.467783 | 4.28% | 2.14% | motif file (matrix) |
| 3 | 0.770 | T A G C A C T G A C G T T A C G C G T A A G T C A C T G G A C T | 1e-19 | -45.742998 | 2.69% | 1.31% | motif file (matrix) |
| 4 | 0.772 | T A C G T A C G G T C A T A G C T C A G A C G T A C T G C G T A A G T C A T C G A G C T G T A C G C T A A T G C | 1e-18 | -43.141703 | 0.46% | 0.04% | motif file (matrix) |
